# NPARS—A Novel Approach to Address Accuracy and Reproducibility in Genomic Data Science

**DOI:** 10.3389/fdata.2021.725095

**Published:** 2021-09-27

**Authors:** Li Ma, Erich A. Peterson, Ik Jae Shin, Jason Muesse, Katy Marino, Matthew A. Steliga, Donald J. Johann

**Affiliations:** ^1^ Winthrop P. Rockefeller Cancer Institute, University of Arkansas for Medical Sciences, Little Rock, AR, United States; ^2^ Department of Information Science, University of Arkansas at Little Rock, Little Rock, AR, United States

**Keywords:** genomics, data science, reproducibility, accuracy, analytic validity

## Abstract

**Background:** Accuracy and reproducibility are vital in science and presents a significant challenge in the emerging discipline of data science, especially when the data are scientifically complex and massive in size. Further complicating matters, in the field of genomic-based science high-throughput sequencing technologies generate considerable amounts of data that needs to be stored, manipulated, and analyzed using a plethora of software tools. Researchers are rarely able to reproduce published genomic studies.

**Results:** Presented is a novel approach which facilitates accuracy and reproducibility for large genomic research data sets. All data needed is loaded into a portable local database, which serves as an interface for well-known software frameworks. These include python-based Jupyter Notebooks and the use of RStudio projects and R markdown. All software is encapsulated using Docker containers and managed by Git, simplifying software configuration management.

**Conclusion:** Accuracy and reproducibility in science is of a paramount importance. For the biomedical sciences, advances in high throughput technologies, molecular biology and quantitative methods are providing unprecedented insights into disease mechanisms. With these insights come the associated challenge of scientific data that is complex and massive in size. This makes collaboration, verification, validation, and reproducibility of findings difficult. To address these challenges the NGS post-pipeline accuracy and reproducibility system (NPARS) was developed. NPARS is a robust software infrastructure and methodology that can encapsulate data, code, and reporting for large genomic studies. This paper demonstrates the successful use of NPARS on large and complex genomic data sets across different computational platforms.

## Introduction

The intersection of data science, analytics, and precision medicine are now having an increasingly important role in the formation and delivery of health care, especially in cancer where the treatment regimens are complex and becoming more individualized (Ginsburg and Phillips, 2018). The National Research Council defined precision medicine as the ability to guide health care toward the most effective treatment for a given patient, improving quality and reducing the need for unnecessary diagnostic testing and therapies (National Research Council, 2011). Our understanding of the genomic basis of disease (cancer) is being transformed by the combination of next generation sequencing (NGS) and state-of-the-art computational data analysis, which are empowering the entry of innovative molecular assays into the clinic, and further enabling precision medicine (Berger and Mardis, 2018). Precision medicine is data science driven (Ginsburg and Phillips, 2018).

*Data science* is a nascent, cross-disciplinary field that can be viewed as an amalgamation of classic disciplines. These include, but are not limited to: statistics, applied mathematics, and computer science, and importantly is focused on finding non-obvious and useful patterns from large datasets (Kelleher and Tierney, 2018). Data science seeks to find patterns and discriminators in order to support actionable decision making (Cao, 2017a; He and Lin, 2020). How can an insight be actionable? Except for domain-specific factors, the *predictive power* of an insight makes itself actionable (Dhar, 2013). A central tenet in science that distinctly extends into data science is *accuracy,* which is the quality or state of being correct or precise. It is also defined as simply the ratio of correctly predicted observations to the total observations, and is utilized to measure predictive power.

Data science is enabling new and different understandings and reshaping several traditional fields (e.g., microbiology and microbiome, supply chain management, astronomy) into heavily data-driven disciplines (Borne, 2010; Hazen et al., 2014; Bolyen et al., 2019). The term “*Data Science*” is becoming increasingly associated with data sets massive in size, but there are additional challenges in this rapidly evolving field. Some factors considered to contribute to the challenges include: 1) *data complexity*, which refers to complicated data circumstances and characteristics, including the quality of data, largeness of scale, high dimensionality, and extreme imbalance; 2) the development of effective algorithms and, common task infrastructures and learning paradigms needed to handle various aspects of data; 3) the appropriate design of experiments; 4) proper translation mechanisms in order to present and visualize analytical results; 5) *domain complexities*, which refers to expert knowledge, hypotheses, meta-knowledge, etc., in the particular subject matter field (Cao, 2017b).

There is a known reproducibility problem in science. This was investigated and quantified by a survey conducted by the journal Nature involving over 1,500 scientists (Baker, 2016). The survey results reported that over 70% of researchers have tried and failed to reproduce another scientist’s results and, more than half have failed to reproduce their own experiments. The survey also uncovered ambiguity concerning the exact definition of reproducibility and, this definition may be different depending on the scientific field.

In data science, *reproducibility* is generally defined as the ability to re-compute data analytic results, with an observed dataset and requisite information regarding the analysis tools (Peng, 2015). Given reproducibility, independent researchers can build up evidence for or in contradiction to a scientific hypothesis (Peng, 2011; Aarts et al., 2015). Some studies have suggested a large number of practical rules or methods for enhancing reproducibility in research (Sandve et al., 2013; Rupprecht et al., 2020). Nonetheless, in several fields, non-reproducibility is still an obstacle towards the better understanding of datasets, further blocking the path to new scientific discoveries (Mobley et al., 2013; Iqbal et al., 2016; Goodman et al., 2018; Wen et al., 2018). In addition, the current situation has forced us to face an awkward truth, that is, while our ability to generate data has grown dramatically, our ability to thoroughly understand data outputs has not developed at the same rate (Peng, 2015). Only if an analytical result is reproducible, can its accuracy be determined. The accuracy itself is based on evaluating the average performance of a series of analytical results from the same dataset. Then can we say such an analytical result is valid and has *analytical validity*. In other words, analytic validity can tell us how well the predictive power of an insight can be. Accuracy and reproducibility are cornerstones of analytical validity.

As more realize the implications and challenges presented by reproducibility in the field of biology, outstanding bioinformatics tools have been developed for improving the situation. To conquer the heterogeneities in bioinformatics tools, Bioconda (Grüning et al., 2018a) integrates more than 3,000 Conda tools. Docker based Dugong (Menegidio et al., 2018) automates the installation of more than 3,500 bioinformatics tools. Pachyderm (Novella et al., 2019) has been developed for managing complicated analyses including multiple stages and multiple tools. For specific studies, reproducible pipelines have been introduced: PiGx (Wurmus et al., 2018) has been created for reproducible genomics analysis, whereas, QIIME 2 (Bolyen et al., 2019) has been released for reproducible, interactive, scalable, and extensible microbiome data science. Finally, many researchers have utilized the web-based platform Galaxy (Jalili et al., 2020) to facilitate collaborative and reproducible (Grüning et al., 2018b) biomedical analyses.

In genomic data science, to address reproducibility, improve scientific accuracy, and enhance collaboration, we present a robust software infrastructure and methodology that can encapsulate data, code, and reporting for large genomic studies. Our system is specifically focused on post-NGS pipeline (downstream) analysis, since it is at this juncture where collaborative endeavors arise focused on gleaning biological insights into studies employing one or more large and complex omics data sets. While the aforementioned tools each offer some methods for tackling the collaborative and reproducibility problems associated with pipeline software, none offer all the features and flexibility in our area of inquiry; post-pipeline (downstream) analysis collaboration and reproducibility. As an example, Galaxy is able to provide collaboration and reproducibility of downstream analyses, however, its ability to execute arbitrary code *via* a programming language of the researcher’s choice—if possible—can be quite burdensome.

**FIGURE 1:**
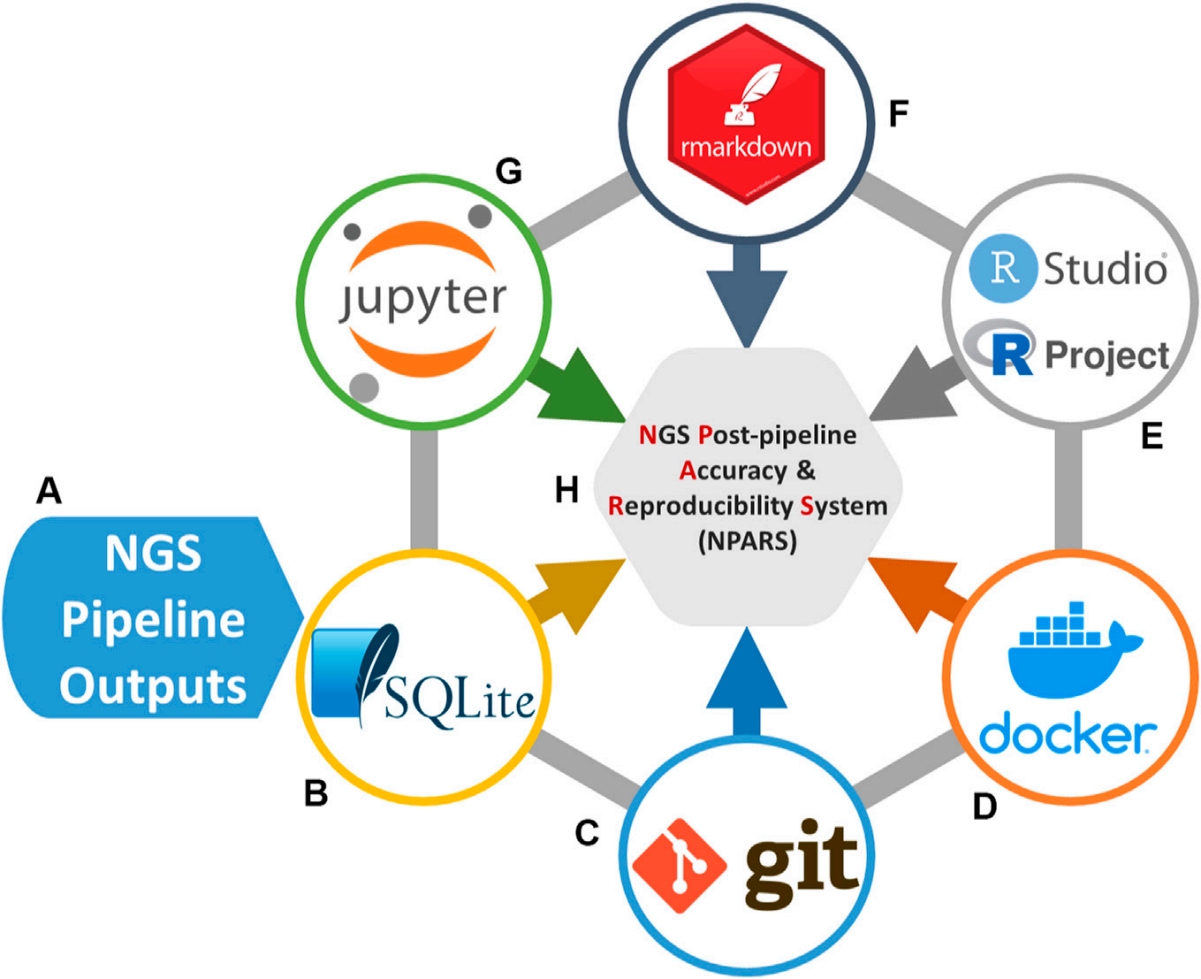
Software technologies used for the NGS Post-pipeline Accuracy and Reproducibility System (NPARS) infrastructure creation. The six core technologies used are shown. **(A)** Study results from a genomics pipeline or repository are extracted and prepared for insertion into a SQLite database. **(B)** SQLite stores all genomic study outputs along with salient study metadata. **(C)** Git provides version control of the Dockerfiles (Docker image specification, i.e., analysis environment) and analysis source code. **(D)** Docker wraps the development environmental information into a container, simplifying software configuration management and, the initialization of a reproducible analysis environment. **(E)** RStudio, provides an integrated development environment for the R programming language and R Projects that are utilized, which provide an efficient way to organize software development activities. **(F)** RMarkdown generates self-documenting analytical reports into HTML files. **(G)** Jupyter Notebooks, are utilized as a development and visualization environment for Python-based projects and reports.

Our system is named NGS Post-pipeline Accuracy and Reproducibility System (NPARS) and its core technologies are graphically illustrated in Figure 1. NPARS is different from other approaches. Specifically, it is the first to focus on the challenges associated with the accuracy, reproducibility, as well as, providing a more convenient manner of collaboration with colleagues. This is achieved by the ability of NPARS to encapsulate large and complex genomic datasets into a portable database container, which may then be analyzed by well-established APIs (Python/Jupyter Notebook, R/Rmd). The infrastructure first loads all data needed for subsequent analyses into a local lightweight (SQLite, 2021) database. The data is then captured within the database along with salient metadata into a schema, which can then be accessed *via* well-known open-source application programming interfaces. These include the use of Jupyter Notebooks (Python) (Kluyver et al., 2016; Python Software Foundation, 2021), RProjects and RMarkdown (R) (Allaire et al., 2021; R-Project, 2021) with an aim to generate self-documenting source code, and results in portable formats. All software may be managed using Docker (Merkel, 2014) containers and Git (Git, 2021) (version control), simplifying configuration management.

## Methods

### Synthetic Data

Synthetic data was used in this study. All synthetic data was derived from actual human tumor tissue data sets (e.g., FastQ files). RNA-seq synthetic data was produced by RSEM (Li and Dewey, 2011). DNA-based synthetic data was produced through aggregation and averaging from a pool of human tumor samples. All FastQ files were initially created from BCL files using bcl2fastq2 v2.18.0.12 (bcl2fastq2 and bcl2fastq, 2021) and when needed or indicated, adapter trimming was performed during the conversion. FastQC v0.11.4 (FastQC, 2021) was used to assess the quality of all FastQ files.

### RNA Sequencing Pipeline

#### Transcriptome Reconstruction and Gene-Level Count Qualification

STAR v2.5.3a (Dobin et al., 2013) was used to align each sample’s paired-end reads to the Ensembl Homo Sapiens reference genome build GRCh37.75, using STAR’s “2-pass” method. Quality control and assessment of resulting BAM files was performed using QualiMap v2.2.1 (García-Alcalde et al., 2012) and STAR output metrics. Picard v2.0.1 (Picard, 2021) was used to add read group information. The marking of duplicate reads and sorting of aligned files was also performed using Sambamba v0.6.5 (Tarasov et al., 2015).

Each sample’s BAM file was initially processed using StringTie v1.3.3b (Pertea et al., 2015), along with Ensembl gene annotations to guide transcriptome reconstruction with novel transcript discovery enabled. Each patient’s samples (i.e., study cohort) transcriptome was merged using StringTie’s merge mode. Finally, the cohort’s BAM files were processed using the newly created merged transcriptome. The StringTie option to output “Ballgown-ready” files was enabled.

Ballgown-ready files containing transcript coverage data was “rolled-up” to the gene-level and the R v4.0.3 (R-Project, 2021) library IsoformSwitchAnalyzeR v1.13.05 (Vitting-Seerup and Sandelin, 2019) was used to disambiguate novel findings from StringTie output. Unnormalized count data was extracted from IsoformSwitchAnalyzeR and used for downstream analysis.

#### RNA Expressed Mutation Calling and Gene Fusion Detection

RNA variants were called using the Broad Institute’s GATK Best Practices for RNA-seq variant calling (Calling Variants in RNAseq, 2021). These steps include the following: STAR was used to align reads to the Ensembl Homo Sapiens reference genome (build GRCh37.75), using the recommended “2-pass” approach. Duplicates were marked and the aligned reads sorted with Sambamba. Next, the tool SplitNCigarReads [GATK v3.9 (McKenna et al., 2010; DePristo et al., 2011)] was used to split reads into exon segments, clip reads which overhang intronic regions, and assign a default MAPQ score of 60 to all reads. Variants were called using the HaplotypeCaller tool (GATK). Gene fusions were detected by passing FastQ files directory to STAR-Fusion v1.4.0 (Haas et al., 2019).

### DNA Sequencing Pipeline

#### Targeted Mutational Panel

FastQ files were submitted to the QIAGEN Data Analysis Center (QIAGEN, 2021) in a tumor/normal configuration and processed using the smCounter2 (Xu et al., 2018) pipeline. The aforementioned pipeline generates aligned reads in BAM format and variants detected in VCF format. Quality control and assessment of resulting BAM files was performed using QualiMap.

#### Low-Pass Whole Genome Copy Number Variation

Each sample’s FastQ paired-end files were aligned to the Ensembl Homo Sapiens reference genome (build GRCh37.75) using BWA v0.7.12 (Li and Durbin, 2009). Quality control and assessment of BAM files was performed with QualiMap. BAM files were post-processed to mark duplicates and sort aligned reads (Sambamba). Copy number data was computational inferred using the R library ichorCNA v0.2.0 (Adalsteinsson et al., 2017).

### Post-pipeline Reproducible Data Science Software Infrastructure

NPARS was implemented using the following software packages: Python v2.7.5/3.7.1; Jupyter Notebooks v6.3.0; IPython v7.22.0 (Pérez and Granger, 2007); R v4.1.0; RStudio v1.4.1717 (RStudio, 2020); RMarkdown v.2.7; SQLite v3.35; Docker v20.10.3; and Git v2.26.2 (Git, 2021).

## Results

### NPARS Overview and Workflow

**FIGURE 2:**
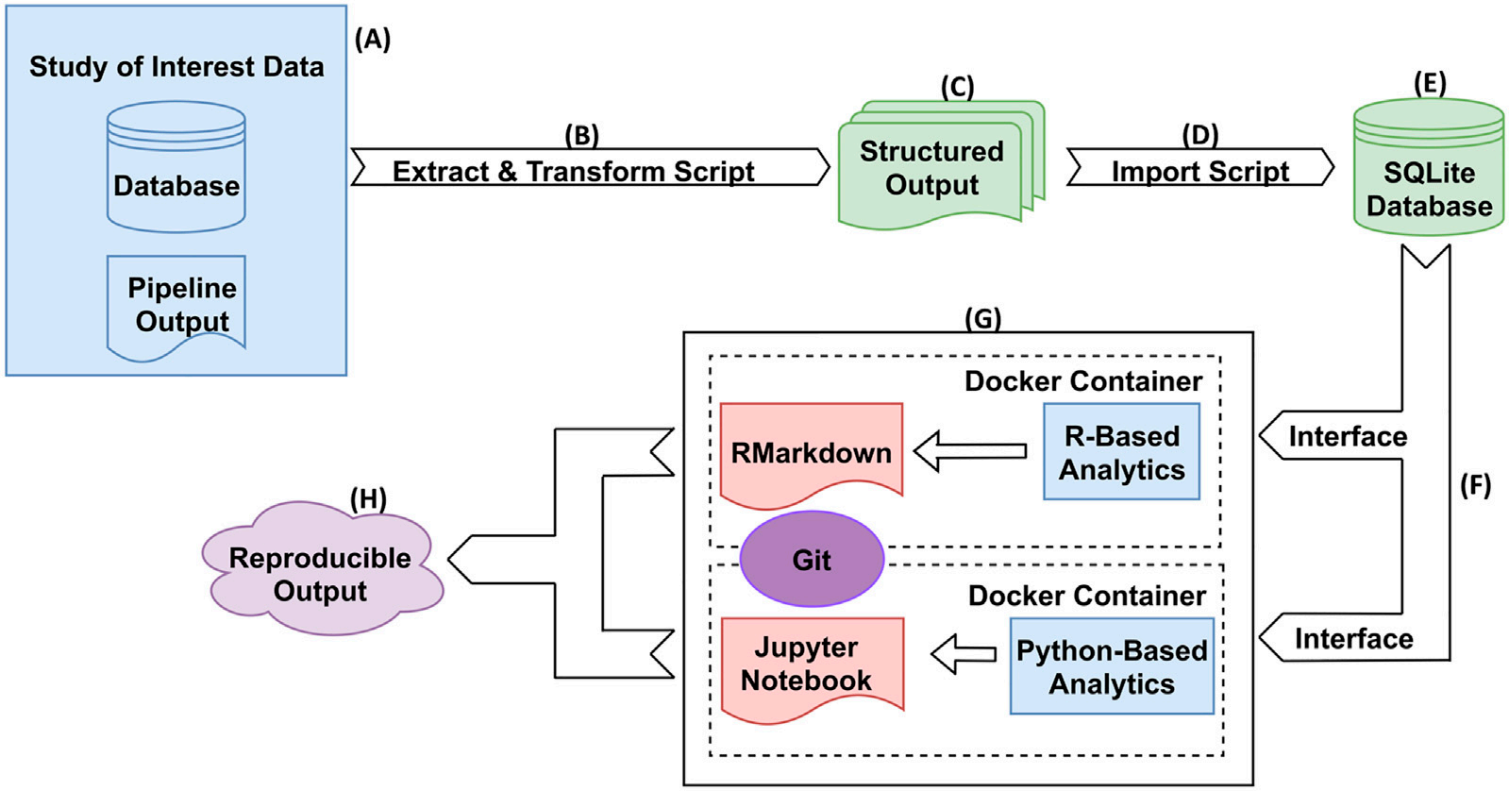
NPARS Overview and Workflow. **(A)** Genomic pipeline output for a particular study of interest is identified. This output can be stored in a database(s) and/or in output files. **(B)** A Python script extracts the identified study results and transforms them into well-defined structured output files. **(C)** The structured output files contain all data and metadata to be imported into the SQLite database. **(D)** A Python script imports the structured output files into the local SQLite database, which already has a well-defined schema to hold the data. **(E)** The SQLite database stores the scientific study data and metadata in a well-organized manner. **(F)** The only interface between the user and the data, is through the particular SQLite API for that development environment. For example, R provides the RSQLite library that provides access to the data. **(G)** Each analysis environment is an abstraction (container) within a Docker container and the source code for it is checked into Git. Self-documenting coding technologies such as R/RMarkdown and Python/Jupyter Notebooks, are used to perform the desired analyses. **(H)** Reproducible reports/analyses are generated, that are both portable and reproducible.

Figure 2 illustrates an overview and workflow for NPARS. First, the data associated with the study of interest is identified. This may be performed from either a central database/repository or directly from pipeline output files as shown in **subfigure (A)**. Next, custom Python scripts are used to perform extraction and transform operations on the pipeline outputs and associated metadata **(B)**. The result is to produce a set of standardized/structured output files, i.e., well-formatted comma-separated files **(C)**. A Python script **(D)** imports the structured output files into the local SQLite database containing a well-defined schema to hold the data. The SQLite database **(E)**, is a light-weighted and easily portable database, and is utilized to store the study’s data and metadata in a well-organized manner. Well known and regarded APIs (RProject and R-Markdown, Jupyter notebooks) are utilized to interface **(F)** to the SQLite database for analysis type activities.

Docker images are utilized to “spin-up” containers, which contain installations of an analysis environment **(G)**. For example, a Docker image containing an R/RStudio environment was created, which includes the necessary libraries (e.g., RMarkdown, DESeq2, etc.) to perform exploratory data analysis (EDA) and differential gene expression on a given study of interest. Python utilizing Jupyter Notebooks is another example analysis environment. Other analysis environments can be easily “Dockerized”, or encapsulate the analysis environment within a Docker image in order to offer the desired functionality. NPARS can also be run without Docker.

Docker image specifications are checked into a Git repository in the Dockerfile format, to allow images to be easily shared and to provide version control of the analysis environments and their dependencies. This greatly aids the ultimate goal of NPARS, which is reproducible output **(H)**. Version controlled analysis source code, can interface directly with a SQLite database *via* well-defined, open-source interfaces provided by the software framework of choice. For example, the R library RSQLite (RSQLite, 2021) may be used to directly query the data to be analyzed from the SQLite database. Finally, given the SQLite database along with access to the Git repository containing the Docker specification and source code, any collaborator may generate a reproducible, complete analysis environment, as well as, analysis results from self-documenting RMarkdown or Jupyter Notebooks.

### Database Schema

**FIGURE 3:**
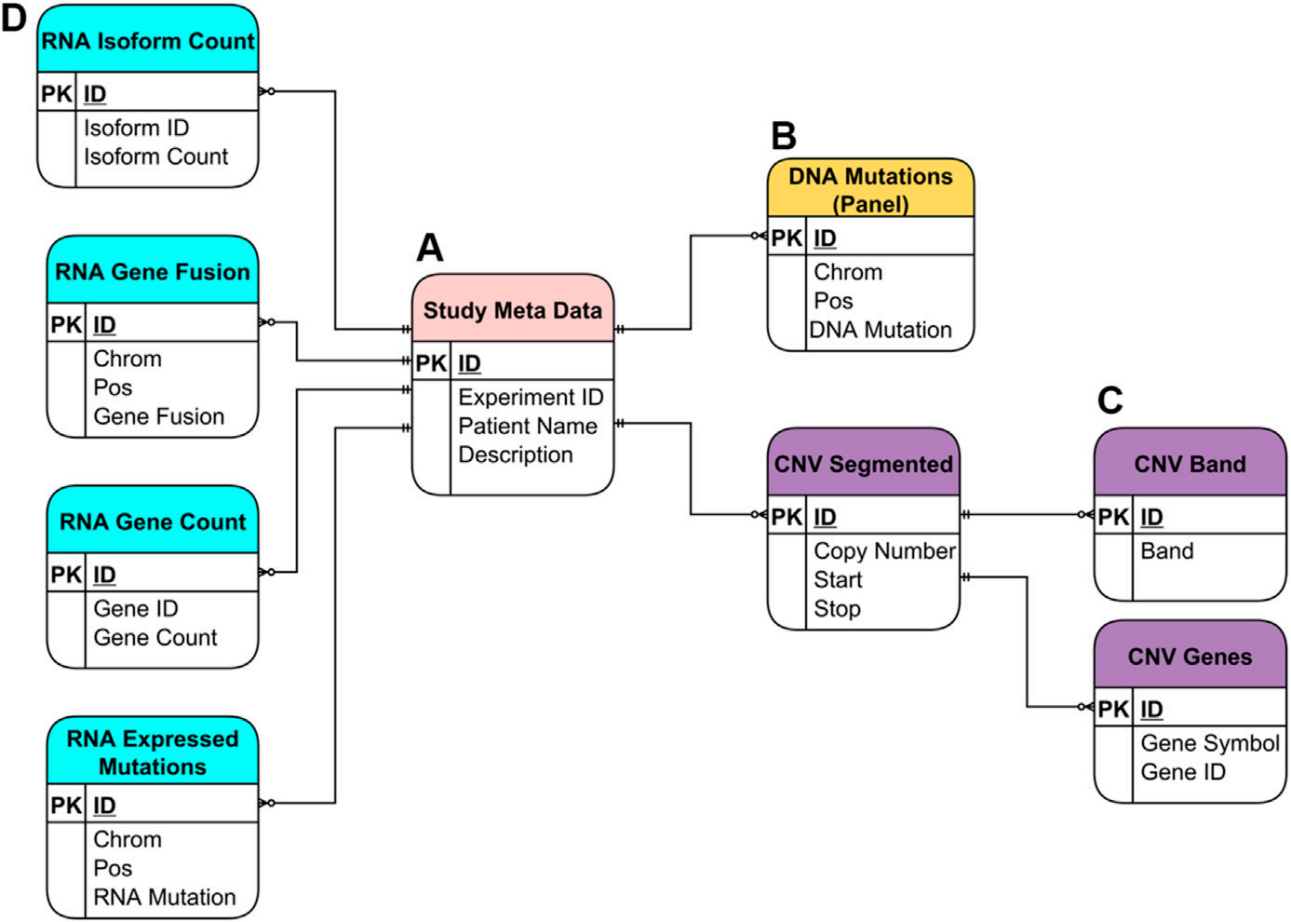
Entity Relationship (ER) Model for the SQLite database utilized in NPARS. Metadata and genomics study data are shown within the context of the database schema. **(A)** Study metadata table (“Study Meta Data”), provides a central repository of metadata, and means of connection to the rest of the tables *via* primary and foreign keys. **(B)** DNA mutations table [“DNA Mutations (Panel)”] contains mutational data from a targeted DNA NGS panel. **(C)** Three tables store copy number variation (CNV) data (“CNV Segmented”), where each CNV segment is a range of chromosome bases of similar copy number value. Each CNV segment is associated with possibly many genes within it (“CNV Genes”), and with possibly many cytobands (“CNV Band”). **(D)** The four tables which hold RNA-based study data: isoform count (“RNA Isoform Count”), gene fusions (“RNA Gene Fusion”), gene count (“RNA Gene Count”) and, expressed mutations (“RNA Expressed Mutations”).

The SQLite database utilized by the NPARS is displayed in Figure 3 and contains several groups of major tables. The entity relationship model illustrates the metadata and genomics study data within the context of the database schema. The *Study Meta Data* table (**subfigure A**) provides an essential repository of metadata, as well as means of central connection to the other database tables *via* a combination of primary and secondary keys. The *DNA Mutations* table (**B**) contains NGS mutational data from a targeted panel.

The remaining tables house six different types of genomic data results. Tables that contain the copy number variation data derived from DNA using an ultra-low-pass whole genome sequencing approach are shown in purple (**C**). As part of the ultra-low-pass approach, copy number data is segmented into chromosomal regions of similar copy number status (*CNV Segmented*) and, each segment/locus is annotated *via* one-to-many relationships with associated genes, (*CNV Genes*) and, associated cytobands (*CNV Band*). Genomic study results include a variety of RNA-based results, which are shown in light blue (**D**). These include isoform count data (*RNA Isoform Count*), gene fusion data (*RNA Gene Fusion*), gene count data (*RNA Gene Count*) which is essentially “rolled up” isoform count data and, expressed mutations data (*RNA Expressed Mutations*).

### Data Analyses

NPARS can generate a wide variety of plots and tables for the purposes of EDA and/or other user-specific analyses, such as finding differentially expressed genes (DEGs). Here we disseminate some examples of reproducible analyses results that were performed on the samples (a total of 21 different NGS experiments yielding large and complex multi-omic datasets), which were described in the Methods section. EDA is an approach for analyzing datasets, summarizing, and showing their main statistical properties in graphics or other data visualization algorithms (Tukey, 1977). Supplementary Figure S1, displays a few examples used in NPARS for RNA-seq data. **Subfigure A** shows violin and box plots displaying the distribution of read counts for the replicates of three classes of samples colored blue, green and maroon. In this example each sample class contains three replicates. Next a principal component analysis plot **(B)** of the samples begins to explore the data. The three tissue types used in this study are circled and color coded. The two principal components explain 72% of the variation. **(C)** A hierarchical clustering analysis (HCA) with heatmap of mean normalized counts, showing the top 20 most variable genes on the *y*-axis, and the three tissue types along with their three replicates colored and listed along the *x*-axis. It is known that tissue types T2 and T3 are biologically similar. Tissue type T1 is known to be biologically different from T2 and T3, and this is reflected in the dendrogram.

In addition to traditional EDA plots, the R library RCircos v.1.2.1 (Zhang et al., 2013) was used in NPARS to visualize multiple NGS studies in a single plot (Supplementary Figure S2). From the outermost ring inward this figure is composed of: **i.** human chromosomal ideogram, **ii.** DNA panel mutations (tumor vs. germline), **iii**. RNA expressed mutations from the full transcriptome, **iv**. whole genome DNA copy number variations (tumor vs. germline) colored according to the legend symbols that denote amplification, normal, or deletion, **v**. RNA gene expression (TPM) and, **vi**. RNA gene fusions.

Differential gene expression (DGE) analysis takes normalized RNA-based read count data and performs a statistical analysis, to find quantitative changes in expression levels between different experimental groups. A DGE analysis report is generated by NPARS, and an abbreviated example output is shown in Supplementary Tables S1, 2. This information was produced as part of a RSQLite query. The novel gene findings report (Supplementary Table S1) is discussed. Subtable **A** shows columns for the following: **i.** predicted novel gene (ID), **ii**. locus, **iii**. gene name corresponding to the nearest annotated gene **iv.** log2 fold change (case over control), **v.** *p*-value, and **vi**. adjusted *p*-value. Subtable **B** displays: **i.** predicted novel gene (ID), **ii.** Case sample mean normalized count (*via* replicates), **iii.** Case sample standard deviation (replicates), **iv.** control sample mean normalized count (replicates) and, **v**. control sample standard deviation (replicates).

Supplementary Table S2 illustrates an abbreviated example report for annotated gene findings. Subtable **A** shows columns for the following: **i.** annotated gene (ID), **ii.** gene symbol, **iii**. locus, **iv.** strand information, **v.** log2 fold change (case over control), **vi.** *p*-value and, **vii**. adjusted *p*-value. Subtable **B** shows columns for the following: **i.** annotated gene (ID), **ii.** Case sample mean normalized count (*via* replicates), **iii**. Case sample standard deviation (replicates), **iv.** control sample mean normalized count (replicates) and, **v.** control sample standard deviation (replicates).

An example of an abbreviated copy number variation (CNV) report derived from an ultra-low-pass whole genome (tumor/germline) NGS approach and processed by the ichor package, was generated by NPARS and is displayed in Supplementary Table S3. The table is produced as part of a RSQLite query and shows columns for the following: **i.** gene symbol, **ii.** annotated gene (ID) per Ensembl, **iii**. Chromosome number, **iv.** Chromosomal segment start position, **v.** chromosomal segment end position, **vi.** median logR, where logR = log2 (T1/Germline), **vii.** subclone status, meaning is the amplication or deletion event part of a subclone per the ichor package **viii.** copy number, **ix.** copy number type and, **x.** cytoband. This report shows a small example of salient CNV findings from a small selection of genes.

A Python/Jupyter Notebook utilizing a library from scikit-learn (Pedregosa et al., 2011) was used to generate the *clustergram* plot in Supplementary Figure S3 by NPARS. This approach is used as part of finding the optimal number of clusters for a K-Means analysis. RNA-seq data normalized across three sample types using DESeq2 were used in this example. The *x*-axis displays the number of clusters (k) during an iteration of k-means clustering analysis, and the *y*-axis displays the PCA weighted mean of the clusters. Each point (red dot) represents the center of a cluster and, the size of each point represents the amount of information contained in each cluster. The thickness of lines (blue) connecting points represent observations potentially moving between clusters. In this example per the clustergram plot the optimal number of clusters should be 2 or 3.

To further investigate the optimal number of clusters for K-Means, *silhouette coefficient plots* (Zhou and Gao, 2014) were performed using the Python/Jupyter Notebook code employing scikit-learn and shown in Supplementary Figure S4. Shown are a series of silhouette plots, which graphically evaluate a variety k-means cluster configurations (2 through 7) along with corresponding silhouette coefficients and threshold value (dotted red vertical line). The value of a silhouette coefficient (*x*-axis) ranges from -1 to 1, the higher the value indicates greater cohesion within the cluster and greater separation between clusters. A negative value indicates a possible improper cluster assignment and, a zero value indicates the object assignment is between clusters. The higher the coefficient value, the more separated and clearly identifiable is the particular cluster. The thickness of each cluster silhouette (*y*-axis, associated with the cluster label) indicates the cluster size. **(A)** Silhouette analysis for k-means clustering on sample data with 2 clusters. **(B)** Silhouette analysis for k-means clustering on sample data with 3 clusters. In this case the new cluster (cluster label 2) has a zero coefficient value meaning it is not significant. **(C)** Silhouette analysis for k-means clustering on sample data with 4 clusters. This plot shows cluster labels 2 and 3 are not significant. **(D)** Silhouette analysis for k-means clustering on sample data with 5 clusters. **(E)** Silhouette analysis for k-means clustering on sample data with 6 clusters. **(F)** Silhouette analysis for k-means clustering on sample data with 7 clusters. According to the plots, the optimal cluster number should be 2. A confluence of evidence based on this evaluation and the previous (clustergram) is indicating the optimal k-means cluster value may be 2. Datasets used to generate the plots are the same simulated data which were used to generate the clustergram plot (Supplementary Figure S3). A Jupyter/Python Notebook was used to perform this analysis.

Based on the prior results from the *clustergram* and *silhouette coefficient plots,* k-means was run twice, once with two clusters, and then three clusters. Supplementary Figure S5 contains results obtained from the Python/Jupyter Notebook code for this analysis, with k-means and two clusters (**A**), and three clusters (**B**). The same RNA-seq data processed by DESeq2 was used. The plot shapes indicate the cluster membership labels: 0, 1, 2 and, the colors represent the tissue types, T1 (Tissue 1, blue), T2 (Tissue 2, orange), T3 (Tissue 3, green). A small red circle is used to highlight the primary difference between the two plots, namely, a new cluster is formed from T1. Analyzing plots **A** and **B**, it appears that two clusters may more efficiently group the data versus three clusters and, supports the results of the *silhouette plots* (Supplementary Figure S4) and, is also in agreement with the *clustergram* plot (Supplementary Figure S3).

## Discussion

The next evolution in oncology research and cancer care are being driven by data science (Yu and Kibbe, 2021). So, it is of paramount importance to address current accuracy and reproducibility issues. In the field of genomic data science, accuracy and reproducibility remains a considerable challenge due to the sheer size, complexity, and dynamic nature plus relative inventiveness of the quantitative biology approaches. The accuracy and reproducibility challenge does not just block the path to new scientific discoveries, more importantly, it may lead to a scenario where critical findings used for medical decision making are found to be incorrect (Huang and Gottardo, 2013). NPARS has been developed to meet the unmet need of improving accuracy and reproducibility in genomic data science. Currently, a limitation of our system is the requirement of the user to put their data into a standardized format for import into NPARS. These steps are not automated.

An accuracy and reproducibility test of NPARS was performed by running the R/RMarkdown and Python Jupyter Notebook code with the SQLite database on two different systems, 1) Windows 10-based system and, 2) system utilizing the Ubuntu Linux distribution. The results demonstrated the use of NPARS on two different systems produced identical outputs and this is summarized in Table 1. Here, the term “Passed” means the observed and expected outputs were identical on the respective systems. The R/RMarkdown outputs were first compared. The RCircos graphic (Supplementary Figure S2), which summarizes and integrates seven genomics studies into a single graphical plot was visually inspected from the Windows and Linux systems and found to be identical. Supplementary Tables S1A,B, 2A,B, 3 were also identical. All EDA graphics from Supplementary Figure S2 were compared by visual inspection and found to be identical. For the analyses performed by Python/Jupyter Notebook, the *clustergram* (Supplementary Figure S3), *silhouette coefficient* plots (Supplementary Figure S4) and k-means graphics (Supplementary Figure S5) were regenerated on each system, compared by close visual inspection and found to be identical.

**TABLE 1:** NPARS Accuracy and Reproducibility Testing Summary.

Analysis test	System #1, Windows 10	System #2, Linux/Ubuntu	Comparative results (system #1 vs. System #2)
RCircos, Supplementary Figure S2	Passed	Passed	Identical
DESeq2 Novel Genes, Supplementary Table S1	Passed	Passed	Identical
DeSeq2 Annotated Genes, Supplementary Table S2	Passed	Passed	Identical
Copy Number Analysis, Supplementary Table S3	Passed	Passed	Identical
Violin Plots, Supplementary Figure S1	Passed	Passed	Identical
Box Plots, Supplementary Figure S1	Passed	Passed	Identical
PCA Plot, Supplementary Figure S1	Passed	Passed	Identical
HCA Plot, Supplementary Figure S1	Passed	Passed	Identical
Clustergram, Supplementary Figure S3	Passed	Passed	Identical
Silhouette Coefficient Plots, Supplementary Figure S4	Passed	Passed	Identical
K-means Plots, Supplementary Figure S5	Passed	Passed	Identical

The first column, “Analysis Test” lists the name of each test along with corresponding supplemental figure or table information. The columns “System #1, Windows-10” and “System #2, Linux/Ubuntu” lists the results of each test run on these respective systems. The column titled “Comparative Results (System #1 vs. System #2) reports the comparative results outcome. The term “Passed” means the observed and expected outputs were the same on the respective systems.

The innovative and evolving landscape of oncology research and cancer care are dependent on accurate, reproducible, and robust data science. High-throughput instrumentation are generating increasingly massive and complex genomic data sets, and continue to create opportunities and challenges in the dynamic field of genomic data science. This makes collaboration, verification, validation, and reproducibility of findings difficult. To address these challenges NPARS was developed. NPARS is the first system to focus on NGS downstream analysis accuracy, reproducibility, and enhancing collaboration, by effectively capturing large and complex genomic datasets into a portable database container and exposing it to well-established APIs. In this paper we have profiled and demonstrated NPARS, which is a robust software infrastructure and methodology that can encapsulate both data, code, and reporting for large genomic studies. This study demonstrates the successful use of NPARS on large and complex genomic data sets across different computational platforms and begins to address the prevailing challenges of accuracy and reproducibility in genomic data science.

## Data Availability

The datasets presented in this study can be found in online repositories. The names of the repository/repositories and accession number(s) can be found below: https://gitlab.com/erichpeterson/npars-analysis.
