# Supplementary material for: NPARS—A Novel Approach to Address Accuracy and Reproducibility in Genomic Data Science
Source: Front Big Data. 2021 Sep 27;4:725095. doi: 10.3389/fdata.2021.725095 (PMC8503682; doi:10.3389/fdata.2021.725095)
Supplement: Supplementary file 1 [file DataSheet1.PDF]

Supplementary Material

1 Supplementary Tables

Supplementary Table 1

(A)

ID	Locus	Nearest Gene	logFC	p-Value	Adjusted p-Value
MSTRG.1030	1:53457613-53457936	SCP2	-1.31	3.95090e-03	3.07194e-02
MSTRG.12247	16:90222055-90222258	RP11-356C4.5	1.63	6.26042e-08	2.44194e-06
MSTRG.12763	17:20689394-20689598	SCDP1	-2.17	4.98764e-05	8.57727e-0
MSTRG.14913	19:7810404-7810975	CD209	3.11	1.12873e-17	2.34340e-15
MSTRG.15749	19:42218890-42265438	CEACAM6	-25.29	9.51938e-11	7.14481e-09

Supplementary Table 1

(B)

ID	Case Mean Normalized Count	Case Std Normalized Count	Control Mean Normalized Count	Control Std Normalized Count
MSTRG.1030	29.02	11.77	74.04	15.53
MSTRG.12247	199.63	13.37	64.02	11.48
MSTRG.12763	19.31	9.48	86.11	12.90
MSTRG.14913	666.33	85.22	77.69	38.83
MSTRG.15749	0.00	0.00	627.30	587.74

Supplementary Table 1. **DESeq2 example report for novel genes produced by the NGS Post-pipeline Accuracy and Reproducibility System (NPARS)**. Subtable **A** shows columns for the following: **i.** predicted novel gene (ID), **ii.** locus, **iii.** gene name corresponding to the nearest annotated gene, **iv.** log2 fold change (case over control), **v.** p-value, and **vi.** adjusted p-value. Subtable **B** displays: **i.** predicted novel gene (ID), **ii.** case sample mean normalized count (via replicates), **iii.** case sample standard deviation (replicates), **iv.** control sample mean normalized count (replicates) and, **v**. control sample standard deviation (replicates). Normalization method used was DESeq2's median of ratios.

Supplementary Table 2

(A)

ID	Gene Symbol	Locus	Strand	logFC	p-Value	Adjusted p-Value
ENSG00000000971	CFH	1:196,652,043-196,747,504	+	1.96	1.53549e-22	5.56757e-20
ENSG00000001036	FUCA2	6:143,494,812-143,511,720	-	0.44	1.71845e-02	9.40871e-02
ENSG00000001460	STPG1	1:24,356,999-24,416,934	-	-1.33	3.79618e-03	2.97410e-02
ENSG00000001626	CFTR	7:117,287,120-117,715,971	+	-3.14	1.75016e-03	1.61550e-02
ENSG00000003137	CYP26B1	2:72,129,238-72,147,862	-	2.21	5.81836e-09	2.88054e-07

Supplementary Table 2

(B)

ID	Case Mean Normalized Count	Case Std Normalized Count	Control Mean Normalized Count	Control Std Normalized Count
ENSG00000000971	23706.62	4791.32	6054.00	867.95
ENSG00000001036	2549.44	532.81	1871.28	149.47
ENSG00000001460	88.67	36.39	224.62	34.96
ENSG00000001626	82.20	80.69	726.53	123.25
ENSG00000003137	619.18	209.97	133.57	1.90

Supplementary Table 2. **DESeq2 example report for annotated genes produced by the NGS Post-pipeline Accuracy and Reproducibility System (NPARS)**. Subtable **A** shows columns for the following: **i.** annotated gene (ID), **ii.** gene symbol, **iii**. locus, **iv.** strand information, **v.** log2 fold change (case over control), **vi.** p-value and, **vii**. adjusted p-value. Subtable **B** shows columns for the following: **i.** annotated gene (ID), **ii.** case sample mean normalized count (via replicates), **iii**. case sample standard deviation (replicates), **iv.** control sample mean normalized count (replicates) and, **v.** control sample standard deviation (replicates). Normalization method used was DESeq2's median of ratios.

Supplementary Table 3

Gene Symbol	Ensembl Gene ID	Chrom	Start	End	Median logR	Subclone	Copy Number	Type	Cytoband
CD274 (PD-L1)	ENSG00000120217	9	1000001	141000000	-0.01	FALSE	2	NEUT	9p24.1
KRAS	ENSG00000133703	12	1000001	132000000	0	FALSE	2	NEUT	12p12.1
MYC	ENSG00000136997	8	1000001	145000000	0	FALSE	2	NEUT	8q24.21
RB1	ENSG00000139687	13	20000001	114000000	0	FALSE	2	NEUT	13q14.2
TP53	ENSG00000141510	17	1000001	81000000	0.01	TRUE	3	GAIN	17p13.1

Supplementary Table 3. Copy number example report by the NGS Post-pipeline Accuracy and Reproducibility System (NPARS). The table shows columns for the following: **i.** gene symbol, **ii.** annotated gene (ID) per Ensembl, **iii**. chromosome number, **iv.** chromosomal segment start position, **v.** chromosomal segment end position, **vi.** median logR, where logR =

log2(T1/Germline), **vii.** subclone status, meaning is the amplification or deletion event part of a subclone per the ichor package **viii.** copy number, **ix.** copy number type and, **x.** cytoband.

2 Supplementary Figures

Supplementary Figure 1

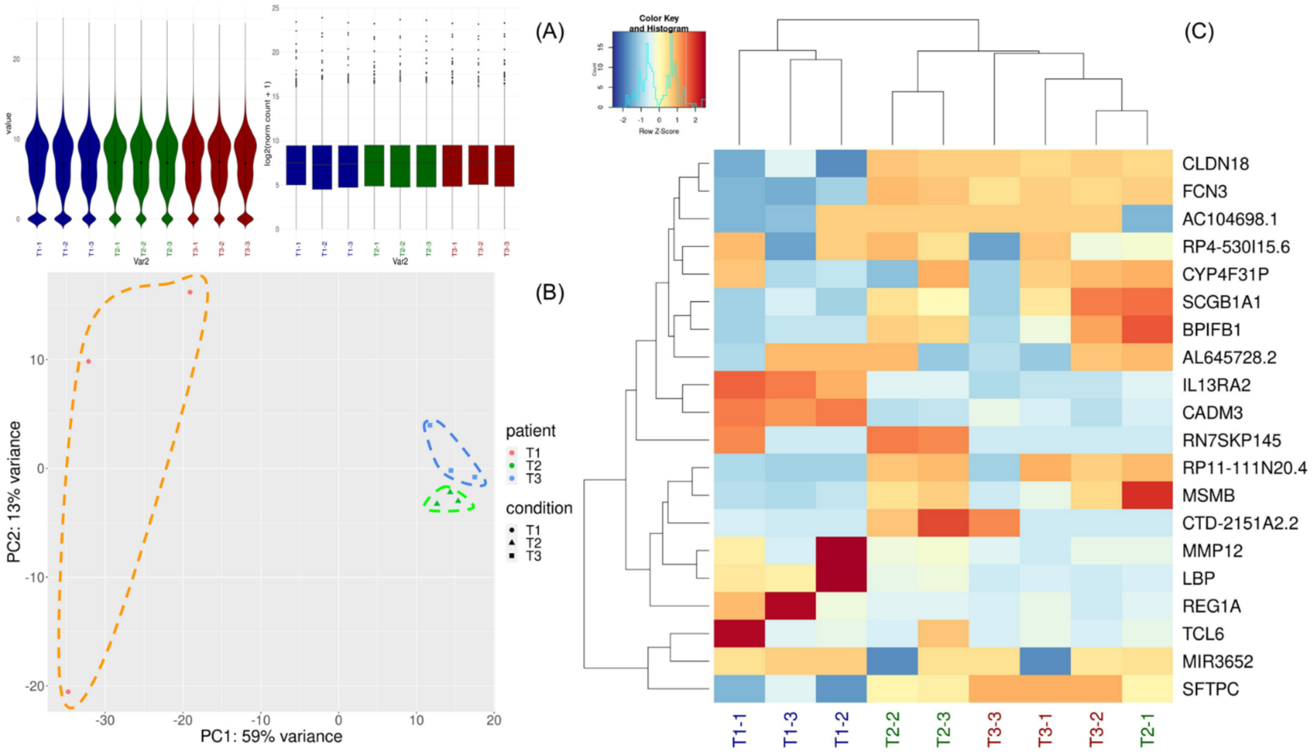

Supplementary Figure 1. Exploratory data analysis plots by the NGS Post-pipeline Accuracy and Reproducibility System (NPARS). **(A)** The violin plot and box plot are used to display the distribution of the read counts of different samples (filtered, normalized (using DESeq2 median of ratios method), log2). **(B)** A principal component analysis (PCA) plot of the samples begins to explore the data (filtered, r-log normalized). **(C)** A hierarchical clustering analysis (HCA) with heatmap of mean r-log normalized counts, showing the top 20 most variable genes on the y-axis, and the samples along the x-axis. R/RMarkdown was used to generate the plots.

Supplemental Figure 2

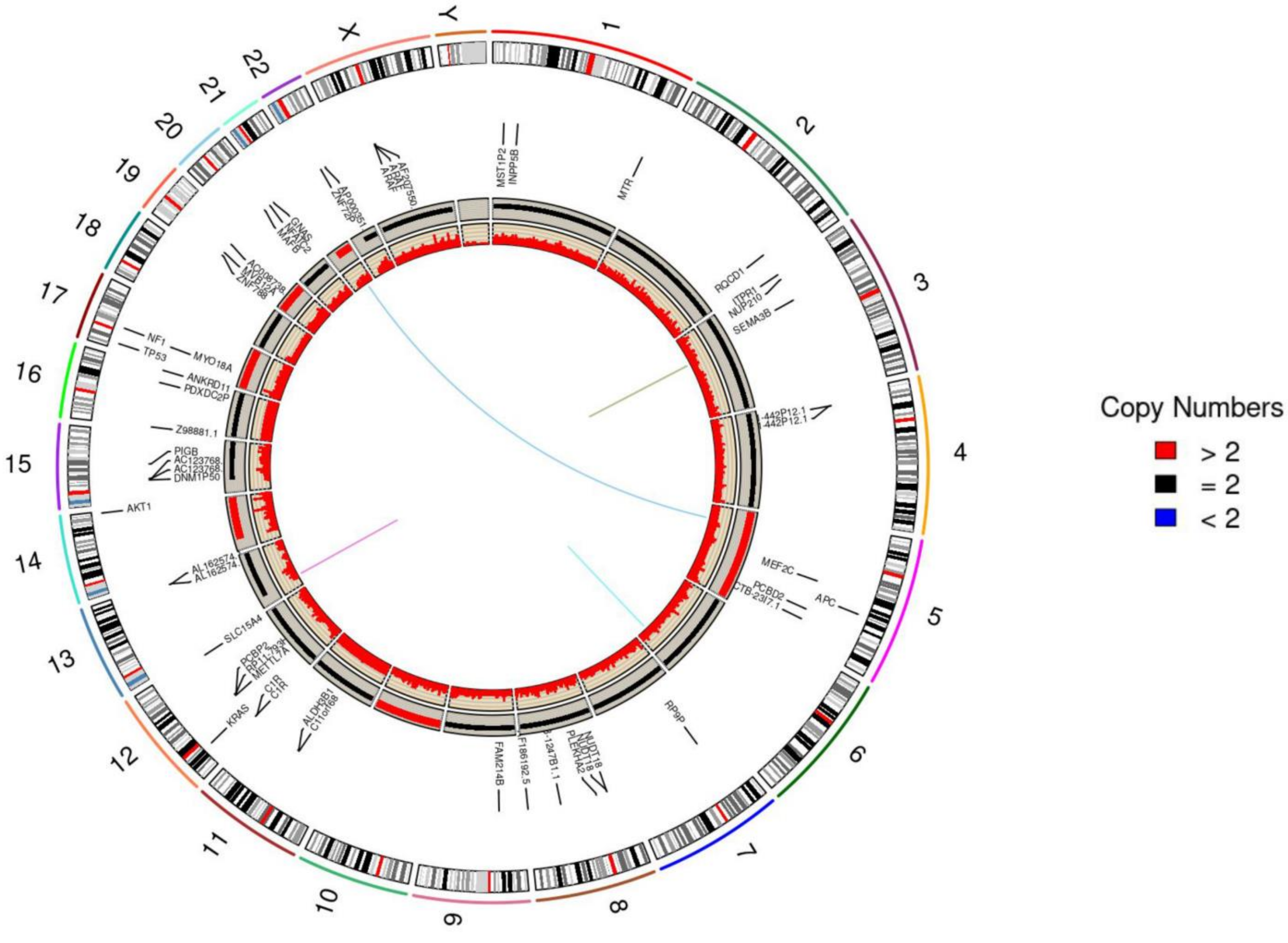

Supplemental Figure 2. RCircos plot produced by NPARS. This figure summarizes and integrates multiple genomics studies into one graphical plot. From the outermost ring inward: **i)** human chromosomal ideogram, **ii)** DNA panel mutations (tumor vs. germline), **iii**) RNA expressed mutations from the full transcriptome, **iv**) whole genome DNA copy number variations (tumor vs. germline) colored according to the legend symbols, **v**) RNA gene expression (TPM) and, **vi**) RNA gene fusions.

Supplementary Figure 3

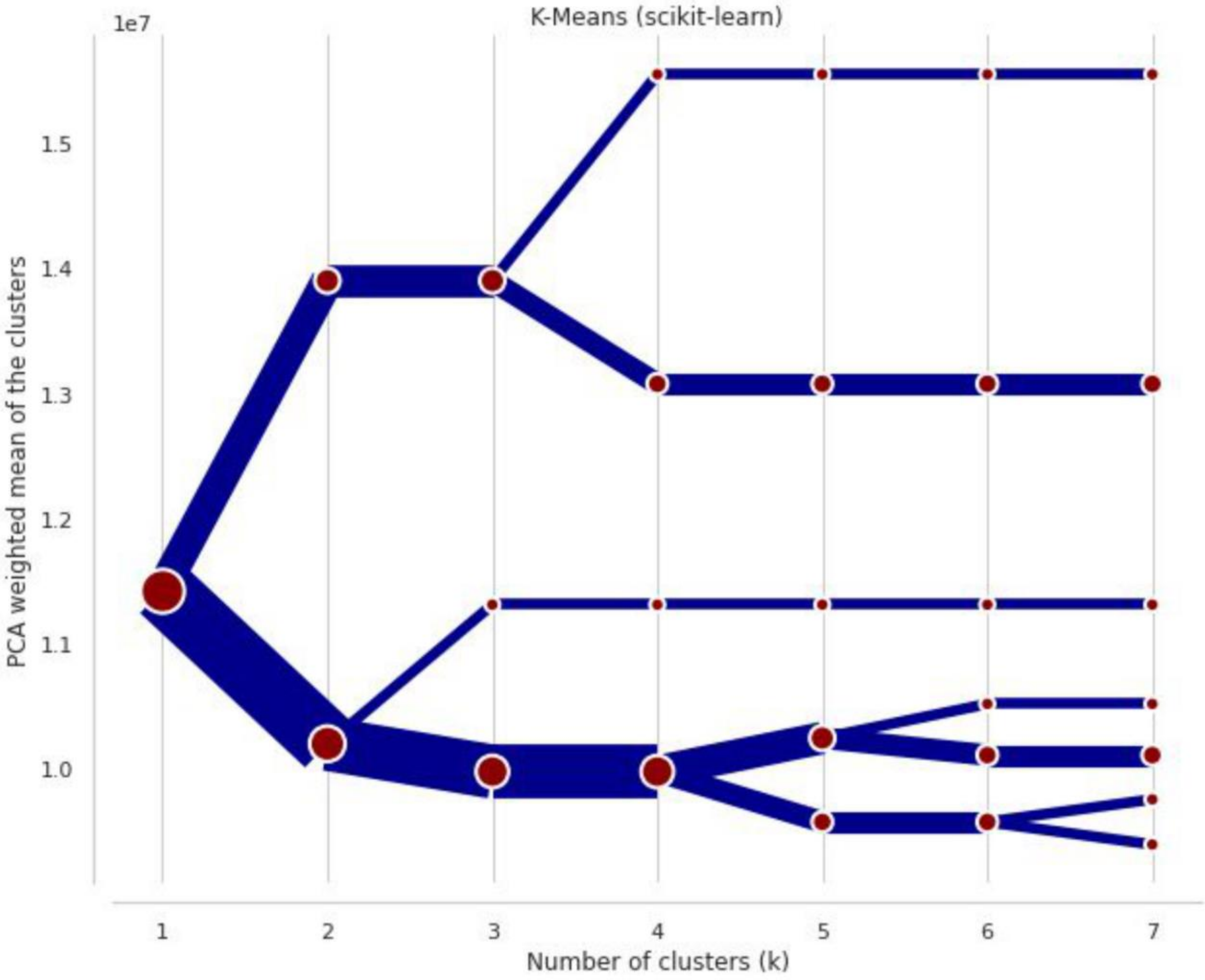

Supplementary Figure 3. Clustergram plot by the NGS Post-pipeline Accuracy and Reproducibility System (NPARS). The x-axis displays the number of clusters (k) during an iteration of k-means clustering analysis, and the y-axis displays the PCA weighted mean of the clusters. Each point represents the center of a cluster. The size of each point represents the amount of information contained in each cluster. The thickness of lines connecting points mean observations moving between clusters. According to the clustergram plot, optimal cluster numbers should be 2 or 3. Dataset used to generate the clustergram plot are simulated cancer genomics data which have been normalized. Python/Jupyter Notebook was used to generate the plot.

Supplementary Figure 4

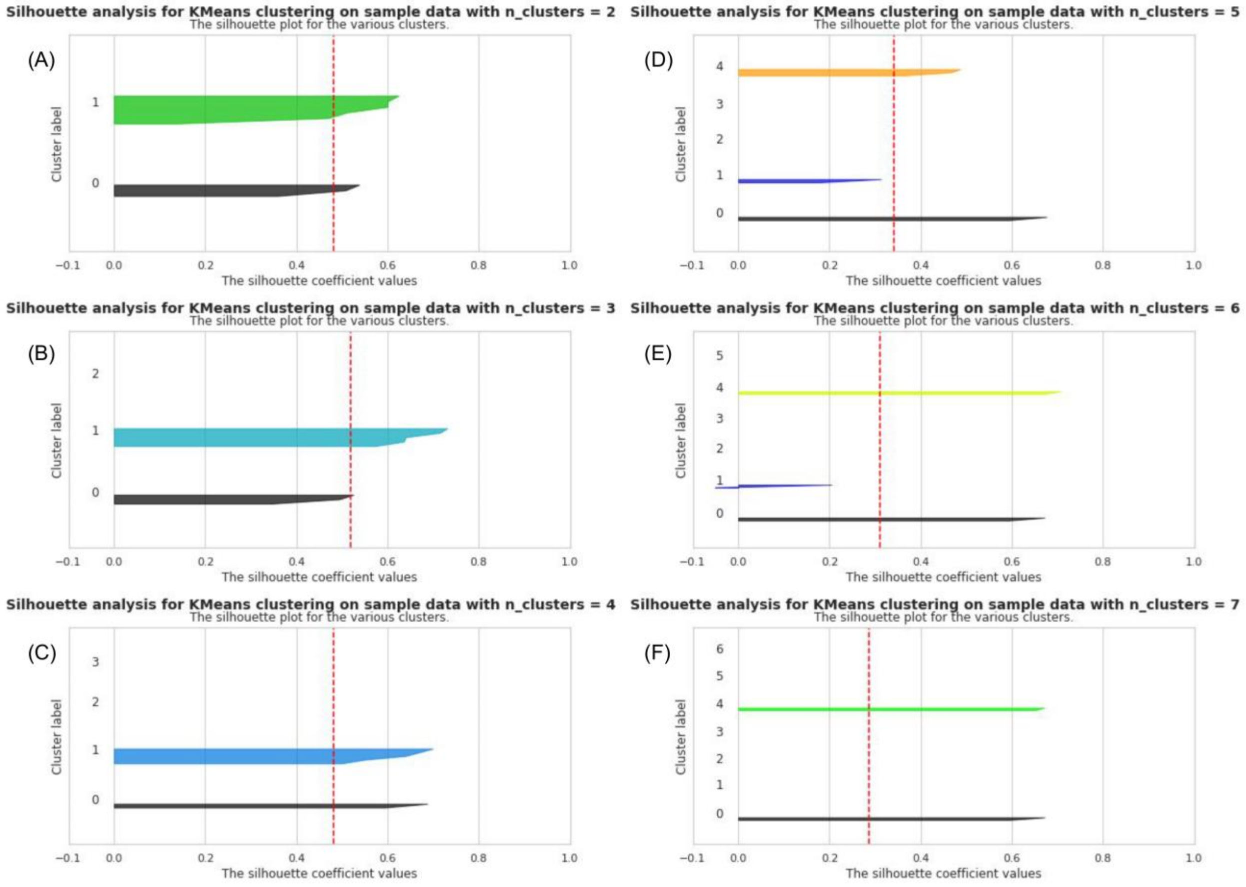

Supplementary Figure 4. Silhouette Coefficient Plots for different cluster numbers by the NGS Post-pipeline Accuracy and Reproducibility System (NPARS). Shown are a series of silhouette plots, which evaluate silhouette coefficients for different numbers of clusters (2 thru 7). The value of a silhouette coefficient ranges from -1 to 1. The higher the value, the more separated and clearly identifiable each cluster is. The thickness of each cluster silhouette indicates the cluster size. **(A)** Silhouette analysis for Kmeans clustering on sample data with 2 clusters. **(B)** Silhouette analysis for Kmeans clustering on sample data with 3 clusters. **(C)** Silhouette analysis for Kmeans clustering on sample data with 4 clusters. **(D)** Silhouette analysis for Kmeans clustering on sample data with 5 clusters. **(E)** Silhouette analysis for Kmeans clustering on sample data with 6 clusters. **(F)** Silhouette analysis for Kmeans clustering on sample data with 7 clusters. According to the plots, optimal cluster number should be 2. Datasets used to generate the plots are the same simulated data which were used to generate the clustergram plot (Supplementary Figure 3). Jupyter/Python was used to generate the plots.

Supplementary Figure 5

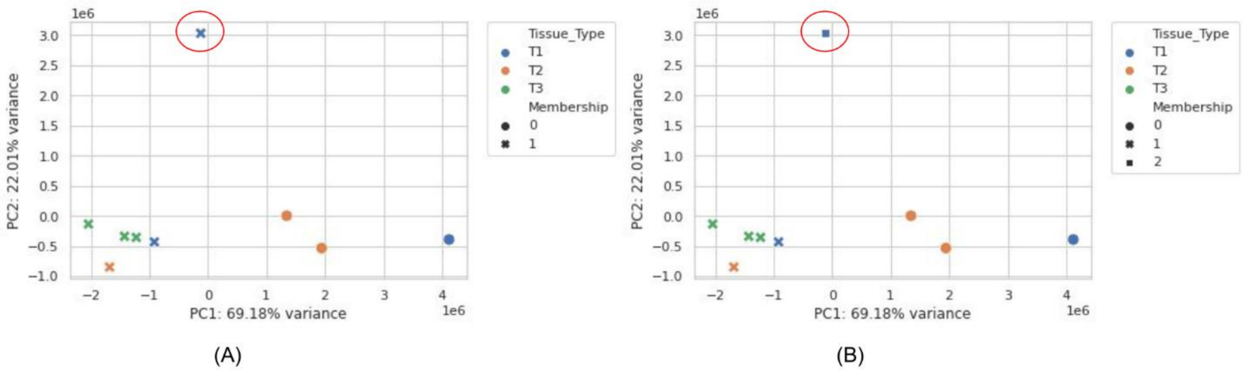

Supplementary Figure 5. K-means Plots for optimal cluster numbers by the NGS Post-pipeline Accuracy and Reproducibility System (NPARS). PCA based K-means clustering plot, using K=2 and K=3. **(A)** K-means plot for 2 clusters (K = 2). **(B)** K-means plot for 3 clusters (K = 3). Plot shapes indicate the cluster labels, 0, 1, 2. Plot colors indicate the tissue type, T1, T2 and T3. According to the two plots, 2 clusters can group the data better than 3 clusters, and the conclusion matches the result of the silhouette plots (Supplementary Figure 4). Datasets used to generate the plots are the same data which were used to generate the Clustergram plot (Supplementary Figure 3) and the silhouette plots (Supplementary Figure 4). Jupyter/Python was used to generate the plot.
